# Analysis of the utilization value of different tissues of *Taxus×Media* based on metabolomics and antioxidant activity

**DOI:** 10.1186/s12870-023-04308-6

**Published:** 2023-05-29

**Authors:** Meng Li, WanRu Geng, Zhi Wang, Qian Wang, Lei Pang, Baoyi Wang, PeiQiang Wang, FengFeng Qu, XinFu Zhang

**Affiliations:** 1 College of Horticulture, Qingdao Agricultural University, Qingdao, 266109 China; 2 Graduate School, University of Jinan, Jinan, 255000 China; 3 Archives, Shandong University of Technology, Zibo, 255049 China

**Keywords:** *T. media*, Widely targeted metabolomics, Metabolites, Differential metabolites, Antioxidant activity

## Abstract

**Background:**

Taxaceae, is a class of dioecious and evergreen plant with substantial economic and ecology value. At present many phytochemical analyses have been performed in *Taxus* plants. And various biological constituents have been isolated from various *Taxus* species. However, the difference of compounds and antioxidant capacity of different tissues of *T. media* is not clear.

**Results:**

In the present study, we investigated the metabolites and antioxidant activity of four tissues of *T. media*, including *T. media* bark (TB), *T. media* fresh leaves (TFL), *T. media* seeds (TS), *T. media* aril (TA). In total, 808 compounds, covering 11 subclasses, were identified by using UPLC-MS/MS. Paclitaxel, the most popular anticancer compound, was found to accumulate most in TS, followed by TB, TFL and TA in order. Further analysis found that 70 key differential metabolites with VIP > 1.0 and p < 0.05, covering 8 subclasses, were screened as the key differential metabolites in four tissues. The characteristic compounds of TFL mainly included flavonoids and tanninsis. Alkaloids and phenolic acids were major characteristic compounds of TS and TB respectively. Amino acids and derivatives, organic acids, saccharides and lipids were the major characteristic compounds of TA. Additionally, based on FRAP and ABTS method, TS and TFL exhibited higher antioxidant activity than TB and TA.

**Conclusion:**

There was significant difference in metabolite content among different tissues of *T. media*. TFL and TS had higher metabolites and antioxidant capacity than other tissues, indicating that TFL and TS were more suitable for the development and utilization of *T. media* in foods and drinks.

**Supplementary Information:**

The online version contains supplementary material available at 10.1186/s12870-023-04308-6.

## Introduction

Taxaceae, is a class of dioecious and evergreen plant with substantial economic and ecology value, and it is mainly distributed in cold temperate and subtropical regions of the Northern Hemisphere [1]. Taxaceae may be divided into 6 extant generas, including over 28 species, such as *T. baccata* L., *T. brevifolia* Nutt., and *T.* × *Media* Rehd [2, 3]. *Taxus*, also known as ‘gold in plants’, is a class of evergreen tree and small shrub and belongs to the family Taxaceae. *Taxus* is widely distributed in many regions of China like western Hubei, Sichuan, Shandong and southern Anhui [4]. Compared to other *Taxus* species, *T.*×*Media* has strong adaptability and fast growth and mainly grows in the United States and Canada. Subsequently, it was introduced and cultivated to China [5].

The *Compendium of Materia Medica* records that *Taxus* can be used to treat cholera, typhoid and other diseases. Modern science proves that the extracts of bark, roots, leaves and arils of *Taxus* have the effect of lowering blood sugar, diuresis, treating kidney disease, menstruating and other diseases [6–8]. *Taxus* is well known for paclitaxel, which is a group of important terpenoid with clinical anticancer efficacy [9]. Paclitaxel can be extracted from the *T. media* whole plant with high and stable content. Typically, paclitaxel can be extracted from the bark of Chinese *taxus* species with a relatively low content [10].Due to the huge medicinal and economic value of paclitaxel, many phytochemical analyses have been performed in *Taxus* plants. And some biological constituents have also been isolated from various *Taxus* species, such as terpenoids, phenols, polysaccharides and flavonoids [11, 12]. These compounds are proved to have strong antioxidant activities, thereby they are as new sources of pharmaceutical drugs against various oxidative stress-induced diseases, like cancer, aging, hyperlipidemia, hyperglycemia, and so on [8, 12–14]. The accumulation levels of biological constituents in different *Taxus* species are different. For example, the level of paclitaxel in *T. yunnanensis* is much higher than *T. fauna* [15]. The fresh twigs of *T. media* accumulates more flavonoids than those of *T. mairei* and *T. cuspidata* [11].

Later research found that the paclitaxel was maily acculmulated in barks and roots of *Taxus chinensis* [16]. The branches and leaves of *Taxus chinensis* and *Taxus madia* × *T*. *Yunnanensis* ‘Yunman’ were important sources for extracting flavonoids and taxol respectively [17, 18]. It can be seen that different tissues of *Taxus* have different potential utilization value. However, until now no study has been done on the comparison of biochemical compositions and antioxidant activities of different issues of *T. media*.

In our study, *T. media*, the most widely distributed in China, was used as experimental material. And four tissues including *T. media* bark (TB), *T. media* fresh leaves (TFL), *T. media* seeds (TS), *T. media* aril (TA) were collected for analyzing metabolites by using ultra-high performance liquid chromatography-tandem and mass spectrometry (UPLC-MS/MS), which has been widely used in metabolomics research recently [3, 19]. Multivariate statistical methods, including principal component analysis (PCA) and Orthogonal partial least squares discrimination analysis (OPLS-DA) were applied to identify the principal differential metabolites. Additionally, we measured the antioxidant activities of different tissues of *T. media*. In this study, we aim to clarify the characteristic compounds and the difference of antioxidant activities of four tissues of *T. media*, which will provide more valuable information for the further development of different issues of *T. media*.

## Materials and methods plant

### Plant materials and treatments

**Fig. 1:**
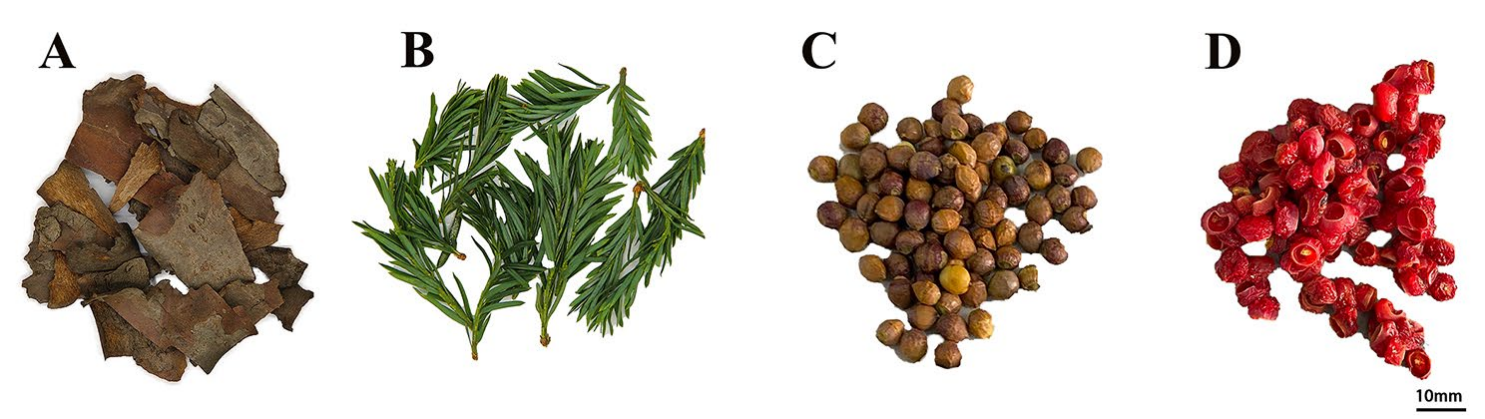
Photographs showing *T. media*. from different tissues. (A) TB, *T. media* bark; (B) TFL, *T. media* fresh leaves; (C) TS, *T. media* seeds; (D) TA, *T. media* aril

In this study, cultivated *T. media* plants were artificially planted in Shandong Zikomandia Taxus Co., Ltd. Zibo, Shandong Province, China. (East longitude 117°55 ‘-118°06’, Northern latitude 36°10 ‘-36°23’). *T. media* bark (TB), *T. media* fresh leaves (TFL), *T. media* arils (TA), and *T. media* seeds (TS) were taken from the 58 years old yew trees with a relatively uniform and good growth (Fig. 1). All fresh samples were snap frozen in liquid nitrogen and stored at -80 ℃ immediately after collecting. The mixture of samples from 10 yew trees was used as a biological replicate. And three replicates were conducted in our study.

### UPLC-MS analysis

#### Sample preparation and extraction

All biological samples were freeze-dried for 24 h in vacuum freeze dryer (Scientz-100 F). Then the samples were grinded into powders by using a grinder (MM400, Retsch, Laichi, Germany); accurately weighed 100 mg of the powder, and extracted with 70% aqueous methanol (1.2 mL), vortex once every 30 min and each lasting 30 s for 6 times in total, placed the mixture in a refrigerator at 4 °C overnight. After centrifugation at 12,000 rpm for 10 min (Sigma, St. Louis, MO, USA), the supernatant fractions were filtrated through a microporous membrane (SCAA-104, 0.22 μm pore size; ANPEL, Shanghai, China) and stored at -80 ℃ for UPLC-MS/MS analysis. Three independent biological replicates were performed for each sample.

### UPLC condition

The extracts of the samples were analyzed using an UPLC-ESI-MS/MS system (UPLC, SHIMADZUNexera X2, Kyoto, Japan; MS, Applied Bio systems 4500 QTRAP, Framingham, MA, USA). An Agilent SB-C18 (1.8 μm×2.1 mm×100 mm) column (Agilent Technologies Corporation, Santa Clara, CA, USA) was used to chromatography separation. Samples were rapidly eluted using solvent A (0.1% formic acid) and solvent B (0.1% formic acid in acetonitrile) were used for both ESI positive and negative modes. Sample measurements were performed using the following gradient program: 0 min, 5% B; 0–9 min, linear gradient increase to 95% B, 9–10 min, 95% B; 10-11.10 min, decrease to 5% B; 11.10–14 min, 5% B. The column oven temperature was set at 40 ℃, flow velocity 0.35 mL/min and the injection volume was 4 µL.

### MS/MS condition

Linear ion trap (LIT) and triple quadrupole (QQQ) scans were acquired on a triple quadrupole-linear ion trap mass spectrometer (QTRAP, API 4500 QTRAP UPLC-MS/MS system) equipped with an ESI turbo ion-spray interface, operating in positive and negative ion modes and controlled by the Analyst 1.6.3 software (AB Sciex, Framingham, MA, USA). The ESI source operation parameters were as follows: ion source, turbo spray; source temperature 550 ℃; and ion spray voltage (IS) 5500 V (positive ion mode)/-4500 V (negative ion mode). In addition, the ion source gas I (GSI), gas II (GSII) and curtain gas (CUR) were set at 55.0 psi, 60.0 psi and 25.0 psi, respectively, and the collision-activated dissociation (CAD) was set at high. Instrument tuning and mass calibration were performed using 10 and 100 µmol/L polpolypropylene glycol solutions in QQQ and LIT modes, respectively. QQQ scans were acquired as (MRM) experiments with a collision gas (nitrogen) pressure of 5 psi. Declustering potential (DP) and collision energy (CE) were performed for individual MRM transitions with further DP and CE optimization. A specific set of MRM transitions were monitored for each period according to the metabolites eluted within this period [20].

### Qualitative and quantitative analysis

In order to compare the contents of each compound in differential samples, the mass spectral peaks in differential samples for each compound were subjected to integration correction on basis of information on retention time and peak type to ensure the accuracy of the qualitative and quantitative analyses. The relative contents of compound in each sample were represented with chromatographic peak area integrals. Three biological replicates were performed for each variety. Compounds were tentatively identified by comparing their primary and secondary MS information, including the accurate precursor ion (Q1) and product ion (Q3) value, retention times (RT), declustering potential (DP), and collision energy (CE) with the self-built database MWDB (Metware Biotechnology Co., Ltd. Wuhan, China), standard products and KNAPSAcK public database (http://kanaya.naist.jp/KNApSAcK) and other the public available metabolite databases [21].

#### Determination of antioxidant activity

Briefly, 100 mg powder were ultrasonically extracted with 10 mL distilled water for 30 min. The samples were centrifuged at 12,000 rpm for 10 min. The supernatant was transferred to a 2 mL centrifuge tube and passed through a 0.22 μm filter membrane and stored in 96-well microplate for Ultra-sensitive multi-function microchannel plate detector analysis. The extracts were used to antioxidant activity analysis.

The FRAP assay was conducted by using a total antioxidant activity assay kit (S0116, Beyotime Biotechnology, Shanghai, China). First, 150 µL TPTZ diluent, 15 uL TPTZ solution and 15 µL detection buffer were added to FRAP solution. Then, 180 µL FRAP solution and 5 µL of the supernatant (distilled water or solution of FeSO_4_ standard) were added to a 96-well microplate. The mixture was mixed and left for 5 min at room temperature under dim light, then the absorbance at 593 nm was measured by using a microplate reader. The results expressed that the concentration of extracts (mmol/g of extracts) had a ferric reducing ability equivalent to that of 1 mM FeSO_4_.

Scavenging capacity of 2,2′-azino-bis-[3-ethylbenzthiazoline-6-sulfonic] (ABTS radical cations) was measured by using a total antioxidant activity assay kit (S0119, Beyotime Biotechnology, Shanghai, China). Briefly, 153 µL detection buffer, 10 µL ABTS solution and 8ul thousandth hydrogen peroxide solution added into ABTS solution. And 170 µL ABTS solution, 20 µL peroxidase solution and 10 µL of the supernatant (distilled water or solution of Trolox standard) were added to a 96-well microplate. The mixture was mixed and left for 5 min at room temperature under dim light, and then the absorbance at 414 nm was measured using a microplate reader. The results were reflected in mmol/g of extracts [15].

### Statistical analysis

The tests were repeated in triplicate, and the result of each test is expressed as the average of three replicates ± standard deviations. The statistically significant differences were tested by the ANOVA using SPSS 17.0 (IBM SPSS, New York, USA). The Venn graphs and Correlation graphs were plotted using R package MetaboAnalystR. SIMCA software (Umetrics, Umea, Sweden) was used to analyze the Principle component analysis (PCA), and orthogonal projections to latent structures-discriminant analysis (OPLS-DA).The metabolites satisfying the following two criteria were selected as differential metabolites: (1) VIP ≥ 1; (2) p-value<0.05. MEV software (Oracle, Redwood shore, USA) was used to compare the difference of metabolites.

## Results

### Analysis of metabolites in TB, TS, TA and TFL

**Fig. 2:**
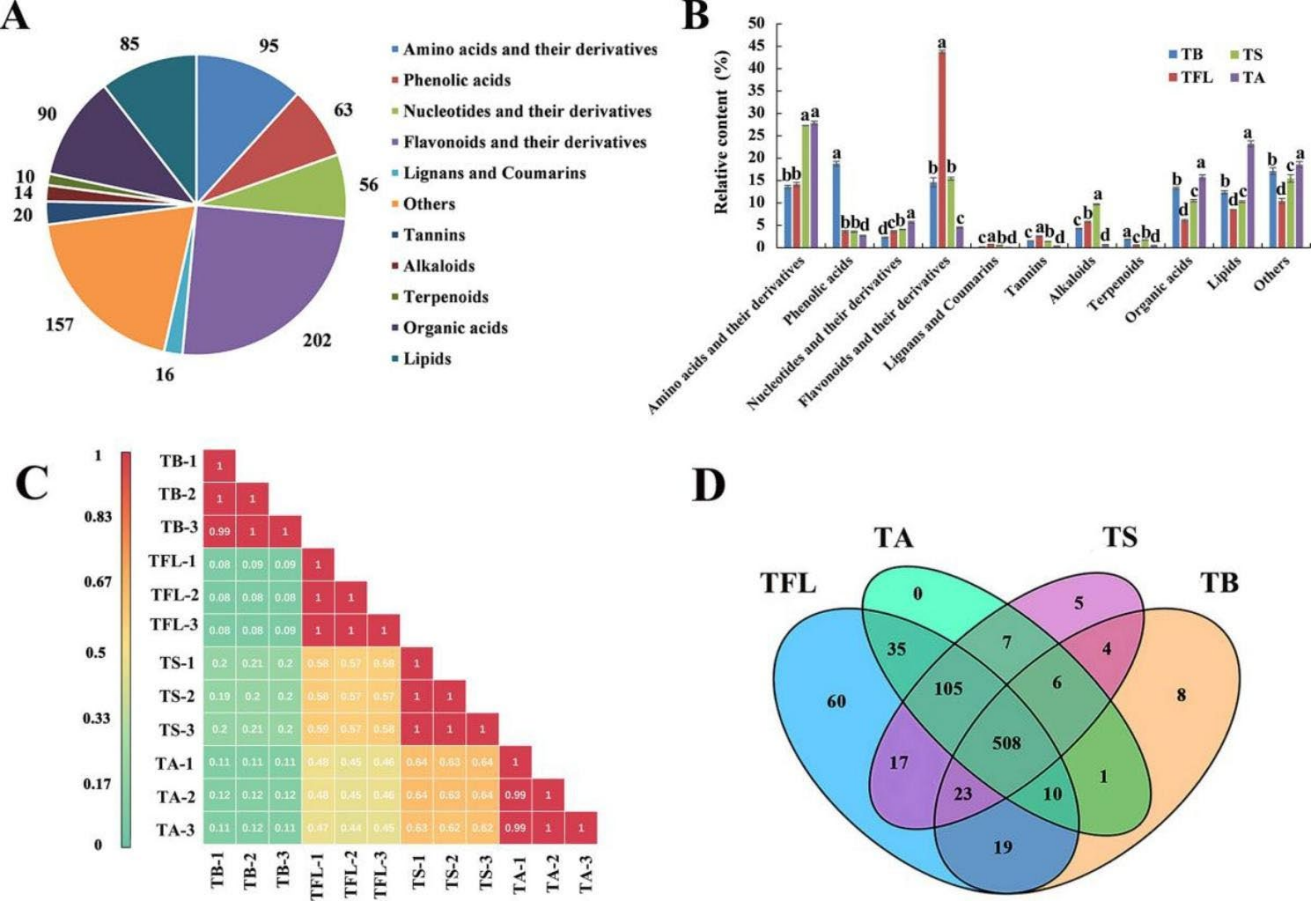
(**A**) The pie chart exhibited the 808 compounds in samples (**B**) The column chart exhibited the 11 compound categories in samples. (**C**) The correlation coefficient in samples. (**D**) The Veen diagram in samples Note: a, b, c and d meant that the same substance has significant difference (*p* < 0.05). Venn diagram showed the overlapping and unique metabolites in samples. TB, *T. media* bark; TFL, *T. media* fresh leaves; TS, *T. media* seeds; TA, *T. media* airls

UPLC-MS/MS was supplied to analyze metabolites in TB, TA, TS and TFL. A total of 808 metabolites were identified by their retention index and mass spectra (Table S1). As shown in Fig. 2A, the metabolites were classified into eleven categories, including 95 amino acids and their derivatives, 63 phenolic acids, 56 nucleotides and their derivatives, 202 flavonoids, 16 lignans and coumarins, 20 tannins, 14 alkaloids, 10 terpenoids, 90 organic acids, 85 lipids and 157 other metabolites (saccharides, alcohols, vitamins and others).

The overlay analysis of the QC-TIC diagram and the samples multi-peak detection diagram (Figure S1) showed that this research data had a good reliability and repeatability. Furthermore, the total amounts of metabolites exhibited the following trend: TFL > TS > TA > TB. The distribution of metabolites in the four tissues of *T. media* weas displayed in Fig. 2B. Eleven types of the metabolites exhibited statistically significant differences (*p* < 0.05) among four differential tissues of *T. media.* It was apparent that the flavonoids, amino acids and their derivatives and phenolic acids were the major groups in *T. media*, which accounted for approximately 50% of the total metabolites. The flavonoids were the dominant part of metabolites in TFL with a relative level of 43.74%, followed by TS, TB and TA. The amino acids and their derivatives also played an important role in biological activity, and the relative percentage was the highest in TA (27.85%), followed by TS, TFL and TB. The content of phenolic acids was the highest in TB (18.79%), about 7 times of TA’s (2.67%). Organic acids and other metabolites were also important in *T. media*. And the relative content of organic acids was in the order of TA > TB > TS > TFL. The content of other metabolites is the highest in TA, followed by TB, TS, TFL. TA had the highest lipids (23.23%), while TFL had the lowest lipids (8.53%). The nucleotides and derivatives, tannins occupied a little proportion in four tissues of *T. media*. Additionally, TB had the highest content of 2,3-dihydroxybenzoic acid (8.16%); TFL had the highest content of avicularin (3.64%); spicataxine was the highest in TS (4.40%). And 3-dehydro-L-threonic acid was the highest in TA (5.16%).

Next, Pearson’s correlation analysis revealed that high correlation coefficients (r = 0.88–1) were observed between the three biological replicates of each sample, demonstrating good sample dependability (Fig. 2C). As shown in the Venn diagram (Fig. 2D), 508 metabolites were common in all samples, which indicated that most metabolites in samples were similar. For instance, the metabolites of TFL, TA and TS had highly consistence, with 105 common metabolites. In addition, 5, 8 and 60 unique metabolites were identified in TS, TB and TFL, respectively, except for TA. More specifically, the unique metabolites of TS included syringaldehyde-4-O-glucoside, kaempferol-3-O-(2-p-coumaroyl) galactoside, biondnoid I, daphnetin, austrotaxine. The unique metabolites of TB included l-Tyramine, 4-aminosalicylic acid, 3,4-dihydroxybenzeneacetic acid, thymine, 5-hydroxy-6,7-dimethoxyflavone, sugiol, citraconic acid, n-(2-hydroxyethyl) eicosapentaenoic acid. l-tyramine, 4-aminosalicylic acid, 3,4-dihydroxybenzeneacetic acid, thymine, 5-hydroxy-6,7-dimethoxyflavone, sugiol, citraconic acid, n-(2-hydroxyethyl) eicosapentaenoic acid. There were 60 unique metabolites identified in TFL, which were distributed in 8 metabolite categories, including kaempferol-4’-O-glucoside, secoisolariciresinol and pinobanksin, naringenin chalcone and other metabolites (Table S3).

### Multivariate statistical analysis

#### PCA analysis

**Fig. 3:**
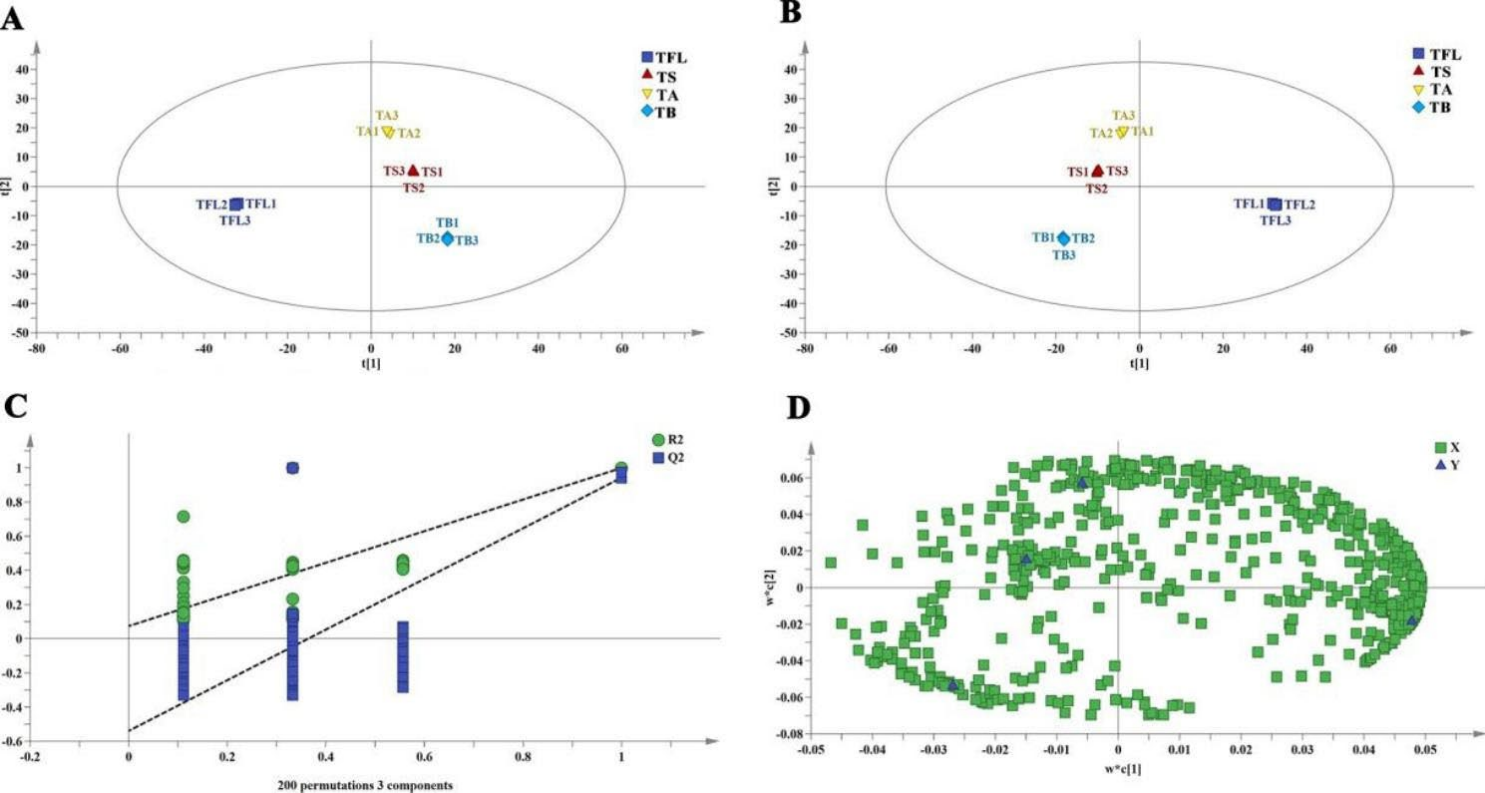
Multivariate statistical analysis based on the whole dataset of metabolites in samples: (**A**) PCA score plot; (**B**) OPLS-DA score plot; (**C**) cross-validation plot of OPLS-DA model with 200 permutation tests; (**D**) OPLS-DA loading plot. TB, *T. media* bark; TFL, *T. media* fresh leaves; TS, *T. media* seeds; TA, *T. media* arils

Based on 808 e metabolites, a non-supervised metrology PCA tool was used to conduct a comprehensive analysis to visualize the difference of metabolites in four samples. In the PCA score plot, two principal metabolites (PC1 and PC2) were constructed and their contribution rates were to be 50.5% and 24.8%, respectively, and the total contribution rate was 75.3%. In the PCA score chart (Fig. 3A), TB, TFL, TS and TA were clearly separated, indicating that the metabolites were significantly different among four tissues. All samples were basically within the 95% confidence intervals and this comparison indicated significant differences in the samples.

#### OPLS-DA analysis

OPLS-DA, a multivariate statistical method for supervised pattern recognition, has better classification and prediction capacity [19]. To find out the key metabolites, the OPLS-DA method was also performed in our study. Figure 3B (t [1] = 50.5%, t [2] = 24.8%) showed that four samples were all within the confidence interval and repeatedly clustered well. TFL was on the right side of this plot, TB was on the lower left, TA and TS were on the upper left, respectively. Thereby, it was indicated that four samples were separated clearly. And there were significant differences in the metabolites between TFL and the other three samples. 200 rounds of random permutations were performed to verify the established OPLS-DA model (R^2^X = 0.967 and Q^2^ = 0.999, respectively). The Q^2^ values exceeded 0.9, indicating that the established OPLS-DA model was remarkable stability and predictive ability (Fig. 3C), and the loading plot was shown in Fig. 3D.

### Differential metabolites screening

The differential metabolites presenting in TB, TFL, TS and TA were further explored. In this study, the metabolites with VIP values ≥ 1 and p < 0.05 were considered as the key differential metabolites. A total of 70 differential metabolites (8 subclasses) were screened, including 19 flavonoids, 10 amino acids and derivatives, 10 organic acids, 7 saccharides and their derivatives, 5 lipids, 4 phenolic acids, 3 alkaloids, and 12 other metabolites. These metabolites were considered as the key differential metabolites to differentiate TB, TFL, TS and TA.

**Fig. 4:**
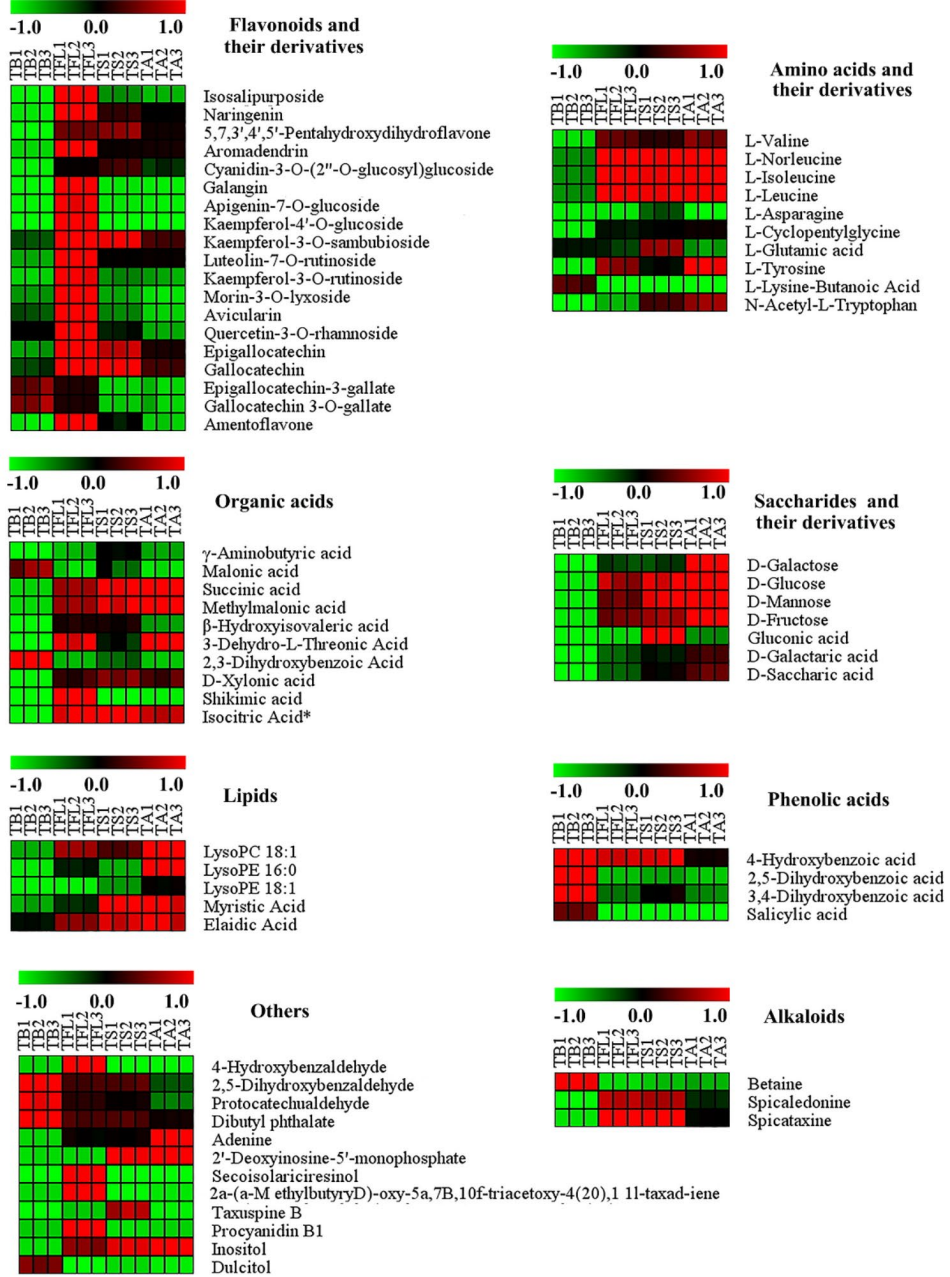
The heat map of the levels of the70 differential metabolites during samples Note: Each sample was represented by a column, and each compound was represented by a row. The abundance of each compound was represented by a bar with specific color. The red color indicates a high abundance of a compound, whereas the green color represents a low relative abundance of a metabolites. TB, *T. media* bark; TFL, *T. media* fresh leaves; TS, *T. media* seeds; TA, *T. media* arils

To observe the differences of metabolites among four samples in a more intuitive manner, a heat map of the 70 differential metabolites was conducted (Fig. 4). The total contents of 10 differential amino acids and their derivatives in different tissues of *T. media* shows the following trend of TA>TFL>TS>TB. The total contents of differential organic acids, saccharides and their deriatives, lipids, nucleotides and their derivatives were highest in TA, followed by TS, TFL, TB in order. The order of the contents of 19 differential flavonoid and their deriatives (naringenin, aromadendrin, galangin, epigallocatechin, gallocatechin etc.) was as follows: TFL>TS>TA>TB. The characteristic compounds of TFL also included procyanidin B1. The contents of differential phenolic acids in different tissues showed the following trend : TB>TS>TFL>TA. Additionally, TB had highest contents of 2,5-dihydroxybenzaldehyde, protocatechualdehyde, dibutyl phthalate and dulcitol. TS had the highest contents of differential alkaloids, followed by TFL, TB and TA in order.

### Antioxidant activity analysis of TB, TS, TA and TFL

The antioxidant is an important indicator of disease prevention. The antioxidant activities of four tissues of *T. media* were compared as shown in Table 1. The antioxidant activities of samples was compared by their total antioxidant activity and ABTS^+^• free radical scavenging activity. Results the total antioxidant activity of TS was the highest, followed by TFL. TB and TA showed the lowest total antioxidant activity with no significance. The ability of ABTS^+^• free radical scavenging of four tissues was TS > TFL > TB > TA. Therefore, our results suggested that TS and TFL showed higher antioxidant activity than TB and TA.

**Table 1:** Antioxidant activities of different samples

	FRAP values(mmol/g)	ABTS values(mmol/g)
TB	0.23 ± 0.01^c^	1.10 ± 0.02^a^
TS	3.44 ± 0.28^a^	0.08 ± 0^c^
TA	0.33 ± 0.01^c^	1.12 ± 0.03^a^
TFL	1.85 ± 0.28^b^	0.14 ± 0.01^b^

Note: Data are presented as mean ± standard deviation (n = 3). Different letters in the same column indicate significant differences between samples (*p* < 0.05). FRAP method: the larger the value is, the stronger the antioxidant activity is; BATS method: the smaller the value is, the stronger the antioxidant activity is. TB, *T. media* bark; TFL, *T. media* fresh leaves; TS, *T. media* seeds; TA, *T. media* arils

## Discussion

In this study, UPLC-MS/MS was applied to identify the differential metabolites in four tissues of *T. media*. In previous studies, Using LC-ESI-Q-TOF-MS approach, 19 metabolites, which were classified as phenolics, flavonoids and terpenes, were tentatively assigned from the *T. chinensis var. maire*i and *T. media* [22]. GC-MS was used to analyze *Taxus Chinensis Var. Mairei* seeds and 24 compounds were identified [23]. Apigenin, luteolin, avicular and other flavonoids were predominantly accumulated in *T. media* [24]. Additionally, a total of 607 metabolites were identified in heartwood and sapwood of *T. chinensi*s and the most abundant metabolites were the flavonoids [25]. Compared with previous studies, our study first analyzed the differential metabolites of four tissues of *T. media* and identified more metabolites. Totally, 808 metabolites were identified, and the amino acids and derivatives, organic acids, flavonoids and phenolic acids, saccharides, alcohols, vitamin and other compounds were found to be abundantly accumulated in *T. media*. In order to clarify the characteristic compounds of different tissues of *T. media*, 70 differential metabolites, covering 8 subclasses, including flavonoids and their derivatives, amino acids and their derivatives, organic acids, saccharides, lipids, phenlolic acids, alkaloids and other compounds were further screened.

In our study, paclitaxel was found to accumulate most in TS, followed by TB, TFL and TA in order (VIP < 1.0; *p* < 0.05). However, paclitaxel was not as the differential compound of four tissues, due to its high contents in TS, TB and TFL. The distribution of paclitaxel in *T. mairei* is significantly affected by tissue differentiation. It is probably the leaves are the major organs for synthesizing paclitaxel precursors, while the barks and roots are the major organs for synthesizing and accumulating paclitaxel [16]. Our results showed except barks, the seeds of *T. media* were also an important ogran for extracting paclitaxel. Furthermore, the high accumulation of paclitaxel in barks was closely related to the expression of ten key enzymes involved in paclitaxel biosythesis [26]. But if the high accumulation of paclitaxel in seeds of *T. media* is also related to the expression of key enzymes involved in paclitaxel biosythesis, it still needs to be further investigated.

Amino acid, as one of the most important macronutrients comprising human diet, play a wide range of physiological functions [27]. In our research, 95 amino acids were examined in four tissues, including 8 essential amino acids like Lys, Trp, Phe, Met, Thr, Ile, Leu and Val. The supplementation of essential amino acids had a beneficial effect on health [28]. The levels of amino acids and derivatives in TS and TA was approximately two times greater than in TB and TFL, which indicate TS and TA were more effective in amino acid supplementation.

Flavonoids have pharmacological activities, such as anti-viral, anti-aging, anti-diabetic and anti-flammatory effects [29]. According to the previous research, flavonoids were identified in twigs, leaves, arils, heartwood and sapwood of variou*s Taxus* species [12, 22, 25]. While in this study, we found the contents of flavonoids, especially epigallocatechin, gallocatechin, naringenin, in TFL were significantly higher than other tissues. We inferred that the synthesis and accumulation of flavonoids mainly occurs in fresh leaves of *T. media*.

Saccharides are one of important energy and flavor subtances, which are widely existing in plants [30]. Our results showed that saccharides and vitamins were found in large percentage in TA, which was consistent with previous study [31]. D-galactose, d-glucose, d-mannose, d-fructose were found to be highly accumulated in TA, and these monosaccharides could be considered as the characteristic compounds of TA. The fruits of Taxus are usually used to make medicinal wine in China [22, 25, 32].

Natural organic acids were widely distributed in most herbs, fruits and vegetables [33, 34]. In our experiment, 90 organic acids metabolites were identified in four tissues. The contents of organic acids exhibited the following trend: TA > TB > TS > TFL. Our research showed that TA contain high proportion of malic acid, citric acid, tartaric acid, oxalic acid and others, which was in line with the previous research results [35, 36]. And these natural organic acids not only are important taste compouds but also have rich bioactivities of antibacterial, hypoglycemic and immune regulation [37]. Our results proved that the TA of *T. media* had relatively high contents of saccharides and organic acids, which could be a good raw material for the production of medicinal wine.

Phenolic acids were found naturally in fruits, vegetables cereals and nuts with bioactive potentials [38–40]. In our results, TB had the highest contents of phenolic acids. And the major characteristic compounds of TB were 4-hydroxybenzoic acid, 2,5-dihydroxybenzoic acid, 3,4-dihydroxybenzoic acid and salicylic acid. Phenolic acids accumulate massively in plant cell walls. And the intermediates generated from phenolic acid metabolism may play active roles in protecting plant from pathogen and/or predator attack, improving cell wall cross links, regulating cell division and expansion, and exerting antioxidant activities [41]. Therefore, the high accumulation of phenolic acids in the barks of T. media may improve the resistance of taxus to external stresses.

In addition, our results showed TFL and TS had stronger total antioxidant activities than TB and TA. It is seemed that the differential metabolites led to the different antioxidant activities of four tissues. The amino acids and derivatives, phenolic acids and flavonoids were significantly accumulated in TFL and TS. Previous studies have shown that these compounds are effective free radical scavengers to prevent healthy cells from being damaged to prevent diabetes, neurodegenerative diseases, and inflammatory diseases [42–44]. Some amino acids and other metabolites play synergistic effect on antioxidant activity [45]. Therefore, we concluded that the above metabolites had a major role in the antioxidant activities of TFL and TS. Based on above data, we deemed that fresh leaves and seeds were more suitable for developing natural antioxidant materials.

## Conclusions

Here, we employed the widely targeted metabolomic analysis to clarified the distribution of metabolites in TB, TFL, TS and TA. Paclitaxel was found to accumulate most in TS, followed by TB, TFL and TA. Totally 70 differential metabolites were screened. 4 phenolic acids, 2 organic acids, 2 flavonoids, 1 amino acid derivative and 1 alkaloid were the major characteristic compounds of TB. The characteristic compounds of TS mainly included 3 organic acids, 2 amino acid derivatives, 2 flavonoids, 1 alkaloid, 1 lipid and 1 saccharide. The key differential compounds of TFL mainly included 15 flavonoids and their derivatives, 2 organic acids, 1 amino acid and 1 alkaloid. 7 alcohols, 6 amino acids, 6 saccharides and their derivatives, 4 lipids and 3 organic acids were the characteristic compounds of TA. Additionally, TFL and TS had higher antioxidant activities than TA and TB. Consequently, our study suggested that the seeds and fresh leaves of *T. media* had enormous development potential in foods and drinks.

## Electronic supplementary material

Below is the link to the electronic supplementary material.

Supplementary Material 1

Supplementary Material 2

Supplementary Material 3

Supplementary Material 4

## Data Availability

All data generated or analyzed during this study are included in this published article.

## Abbreviations

TB: *T. media* bark
TFL: *T. media* fresh leaves
TS: *T. media* seeds
TA: *T. media* arils
Q TRAP: Triple quadrupole linear ion trap
LIT: Linear ion trap
QQQ: Triple quadrupole
QC: Quality control
IS: Ion spray voltage GSI:Gas I
GSII: Gas II
CUR: Curtain gas
CAD: Collision gas
MRM: Multiple reaction monitoring
DP: Declustering potential
CE: Collision energy
TIC: Tent analysis
PCA: Principal component analysis
OPLS-DA: Partial least-squares discriminant analysis
