# Supplementary material for: Analysis of the utilization value of different tissues of Taxus×Media based on metabolomics and antioxidant activity
Source: BMC Plant Biol. 2023 May 29;23:285. doi: 10.1186/s12870-023-04308-6 (PMC10226233; doi:10.1186/s12870-023-04308-6)
Supplement: Supplementary file 1 — Supplementary Material 1 [file 12870_2023_4308_MOESM1_ESM.docx]

**Supplementary Figure 1.**


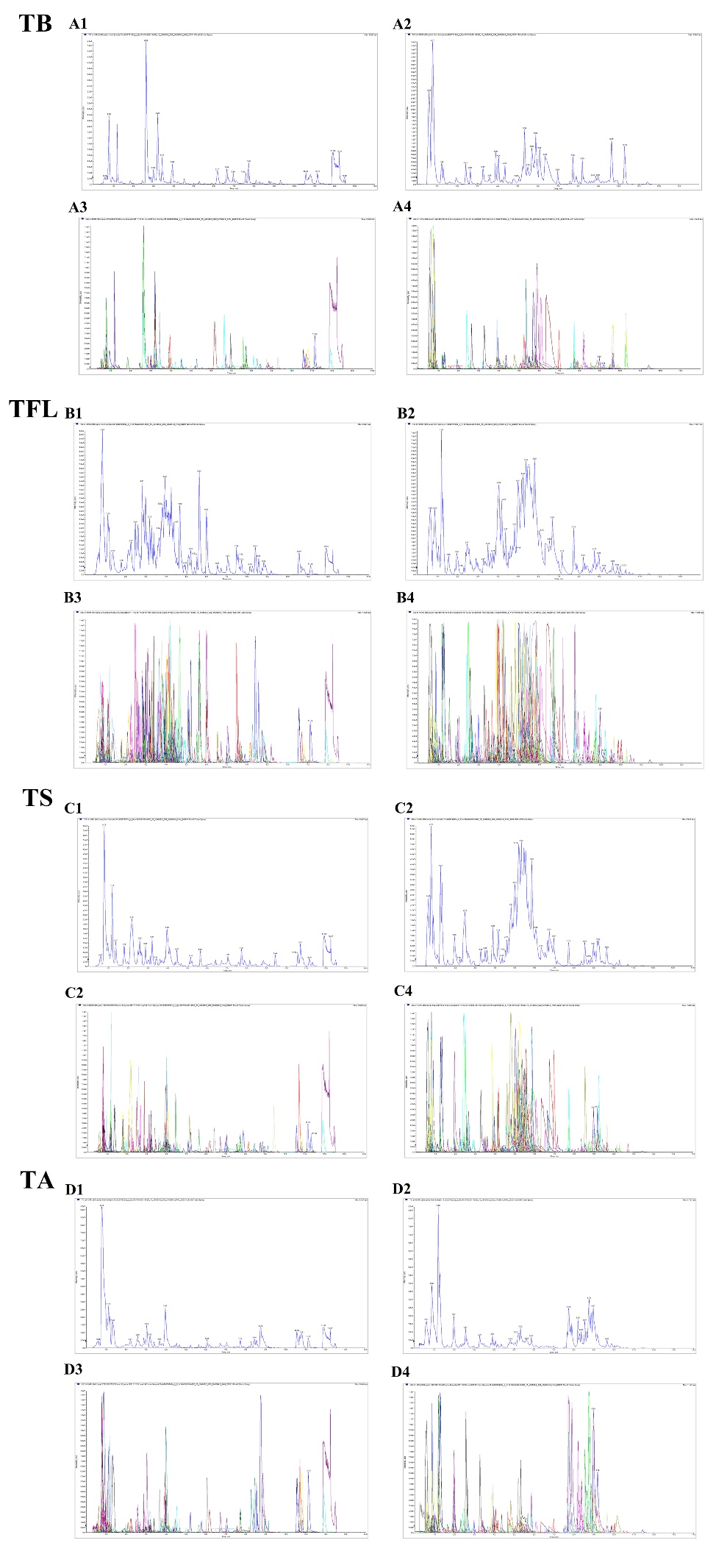


**Figure S1.** The total ion chromatograms of all samples.

**Note:** Total ion current of one quality control sample by mass spectrometry detection (**A1, A2, B1, B2, C1, C2, D1, D2)** and multi-peak detection plot of metabolites in the multiple reaction monitoring mode (**A3** **A4, B3, B4, C3, C4, D3, D4**). **A1, A3, B1, B3, C1, C3, D1** and **D3** were acquired in positive ionization mode. **A2**, **A4, B2, B4, C2, C4, D2** and **D4** were acquired in negative ionization mode.
