# Supplementary material for: Analysis of the utilization value of different tissues of Taxus×Media based on metabolomics and antioxidant activity
Source: BMC Plant Biol. 2023 May 29;23:285. doi: 10.1186/s12870-023-04308-6 (PMC10226233; doi:10.1186/s12870-023-04308-6)
Supplement: Supplementary file 3 — Supplementary Material 3 [file 12870_2023_4308_MOESM3_ESM.docx]

**Table S2. The unique metabolites of all samples**

| compounds | Class | Formula | Molecular Weight | Relative content (%) |
| --- | --- | --- | --- | --- |
| TS | | | | |
| Syringaldehyde-4-O-glucoside | Others | C_15_H_20_O_9_ | 344.111 | 0.01±0.00 |
| Kaempferol-3-O-(2''-p-Coumaroyl)galactoside | Flavonoids and  their derivatives | C_30_H_26_O_13_ | 594.137 | 0.21±0.00 |
| Biondnoid I | Flavonoids and  their derivatives | C_30_H_26_O_13_ | 594.137 | 0.00±0.00 |
| Daphnetin | Lignans and Coumarins | C_9_H_6_O_4_ | 178.027 | 0.08±0.01 |
| Austrotaxine | Alkaloids | C_41_H_53_NO_13_ | 767.351 | 0.05±0.00 |
| TB | | | | |
| L-Tyramine | Amino acids and  their derivatives | C_8_H_11_NO | 137.084 | 0.05±0.03 |
| 4-Aminosalicylic acid | Phenolic acids | C_7_H_7_NO_3_ | 153.043 | 0.01±0.01 |
| 3,4-Dihydroxybenzeneacetic acid | Phenolic acids | C_8_H_8_O_4_ | 168.042 | 0.11±0.12 |
| Thymine | Nucleotides and  their derivatives | C_5_H_6_N_2_O_2_ | 126.043 | 0.00±0.00 |
| 5-Hydroxy-6,7-dimethoxyflavone | Flavonoids and  their derivatives | C_17_H_14_O_5_ | 298.084 | 0.05±0.03 |
| Sugiol | Terpenoids | C_20_H_28_O_2_ | 300.209 | 0.16±0.11 |
| Citraconic acid | Organic acids | C_5_H_6_O_4_ | 130.027 | 0.01±0.01 |
| N-(2-Hydroxyethyl)eicosapentaenoic acid | Lipids | C_22_H_37_NO_3_ | 363.277 | 0.01±0.01 |
| TFL | | | | |
| 2,3-Dimethylsuccinic acid | Amino acids and  their derivatives | C_6_H_8_O_4_ | 144.042 | 0.01±0.00 |
| Cyclo(Pro-Pro) | Amino acids and  their derivatives | C_10_H_14_N_2_O_2_ | 194.106 | 0.01±0.00 |
| Benzaldehyde | Organic acids | C_7_H_6_O | 106.041 | 0.02±0.00 |
| o-Anisic acid | Phenolic acids | C_8_H_8_O_3_ | 152.047 | 0.01±0.00 |
| Homovanillic acid | Phenolic acids | C_9_H_10_O_4_ | 182.058 | 0.00±0.00 |
| Sinapoylglucuronic acid | Phenolic acids | C_17_H_20_O_11_ | 400.101 | 0.37±0.02 |
| p-Coumaroylcaffeoyltartaric acid | Phenolic acids | C_22_H_18_O_11_ | 458.085 | 1.29±0.08 |
| Quillaic acid | Phenolic acids | C_30_H_46_O_5_ | 486.335 | 0.00±0.00 |
| Naringenin chalcone* | Flavonoids and  their derivatives | C_15_H_12_O_5_ | 272.068 | 0.00±0.00 |
| Sieboldin | Flavonoids and  their derivatives | C_21_H_24_O_11_ | 452.131 | 0.28±0.01 |
| 4',6-Dihydroxy-5,7-dimethoxyflavanone | Flavonoids and  their derivatives | C_17_H_16_O_6_ | 316.095 | 0.01±0.00 |
| Eriodictyol-7-O-Rutinoside (Eriocitrin) | Flavonoids and  their derivatives | C_27_H_32_O_15_ | 596.174 | 0.15±0.02 |
| Pinobanksin | Flavonoids and  their derivatives | C_15_H_12_O_5_ | 272.068 | 0.20±0.03 |
| Malvidin-3-O-glucoside (Oenin)* | Flavonoids and  their derivatives | C_23_H_25_O_12_+ | 493.134 | 0.00±0.00 |
| Malvidin-3-O-galactoside (Primulin)* | Flavonoids and  their derivatives | C_23_H_25_O_12_+ | 493.134 | 0.00±0.00 |
| Cyanidin-3-O-(6''-O-p-coumaroyl)sophoroside-7-O-glucoside | Flavonoids and  their derivatives | C_42_H_47_O_23_+ | 919.25 | 0.00±0.00 |
| 3',4',7-Trihydroxyflavone | Flavonoids and  their derivatives | C_15_H_10_O_5_ | 270.053 | 0.01±0.00 |
| Robinetin | Flavonoids and  their derivatives | C_15_H_10_O_7_ | 302.043 | 0.00±0.00 |
| Tricetin (5,7,3',4',5'-Pentahydroxyflavone) | Flavonoids and  their derivatives | C_15_H_10_O_7_ | 302.043 | 0.01±0.00 |
| 2-Glucosyloxy-(4-hydroxyphenyl)acetic acid (Dhurrin acid) | Flavonoids and  their derivatives | C_14_H_18_O_9_ | 330.095 | 0.01±0.00 |
| Acacetin-7-O-galactoside | Flavonoids and  their derivatives | C_22_H_22_O_10_ | 446.121 | 0.11±0.00 |
| Kaempferol-4'-O-glucoside* | Flavonoids and  their derivatives | C_21_H_20_O_11_ | 448.101 | 0.01±0.00 |
| Chrysoeriol-5-O-glucoside | Flavonoids and  their derivatives | C_22_H_22_O_11_ | 462.116 | 0.00±0.00 |
| Eupatilin-7-O-glucoside | Flavonoids and  their derivatives | C_24_H_26_O_12_ | 506.142 | 0.00±0.00 |
| Apigenin-7-O-(6''-p-Coumaryl)glucoside | Flavonoids and  their derivatives | C_30_H_26_O_12_ | 578.142 | 0.21±0.01 |
| Chrysoeriol-7-O-rutinoside | Flavonoids and  their derivatives | C_28_H_32_O_15_ | 608.174 | 0.39±0.01 |
| Myricetin-3-O-galactoside-3'-O-rhamnoside* | Flavonoids and  their derivatives | C_27_H_30_O_17_ | 626.148 | 0.00±0.00 |
| Kaempferol-6,8-di-C-glucoside-7-O-glucoside | Flavonoids and  their derivatives | C_33_H_40_O_21_ | 772.207 | 0.00±0.00 |
| 3-Hydroxyflavone | Flavonoids and  their derivatives | C_15_H_10_O_3_ | 238.063 | 0.00±0.00 |
| Quercetin-3-O-(6''-malonyl)galactoside | Flavonoids and  their derivatives | C_24_H_22_O_15_ | 550.096 | 0.04±0.01 |
| Isorhamnetin-3-O-galactoide-7-O-rhamnoside | Flavonoids and  their derivatives | C_28_H_32_O_16_ | 624.169 | 0.01±0.00 |
| Myricetin-3-O-rutinoside* | Flavonoids and  their derivatives | C_27_H_30_O_17_ | 626.148 | 1.25±0.04 |
| Quercetin-3-O-xylosyl(1→2)glucosyl(1→2)glucoside | Flavonoids and  their derivatives | C_32_H_38_O_21_ | 758.191 | 0.00±0.00 |
| 6-Hydroxykaempferol-3-O-rutin-6-O-glucoside | Flavonoids and  their derivatives | C_33_H_40_O_21_ | 772.206 | 0.00±0.00 |
| Gossypetin-3-O-rutinoside-8-O-rhamnoside | Flavonoids and  their derivatives | C_33_H_40_O_21_ | 772.206 | 0.02±0.00 |
| Quercetin-3-O-rutinoside-7-O-glucoside | Flavonoids and  their derivatives | C_33_H_40_O_21_ | 772.206 | 0.00±0.00 |
| Isorhamnetin-3-O-sophoroside-7-O-rhamnoside | Flavonoids and  their derivatives | C_34_H_42_O_21_ | 786.222 | 0.00±0.00 |
| 6-Hydroxykaempferol-3,7,6-O-triglycoside | Flavonoids and  their derivatives | C_33_H_40_O_22_ | 788.201 | 0.00±0.00 |
| Quercetin-3-O-(2'''-p-coumaroyl)sophoroside-7-O-glucoside | Flavonoids and  their derivatives | C_42_H_46_O_24_ | 934.238 | 0.00±0.00 |
| Hispidulin-8-C-(2''-O-xylosyl)xyloside | Flavonoids and  their derivatives | C_26_H_28_O_14_ | 564.148 | 0.09±0.00 |
| 8,8'-Methylenebiscatechin | Flavonoids and  their derivatives | C_31_H_28_O_12_ | 592.158 | 0.01±0.00 |
| Catechin-catechin-catechin | Flavonoids and  their derivatives | C_45_H_38_O_18_ | 866.206 | 0.00±0.00 |
| Genistein | Flavonoids and  their derivatives | C_15_H_10_O_5_ | 270.053 | 0.01±0.00 |
| Daidzein-4'-O-glucoside | Flavonoids and  their derivatives | C_21_H_20_O_9_ | 416.111 | 0.03±0.00 |
| 6''-O-Acetylgenistin | Flavonoids and  their derivatives | C_23_H_22_O_11_ | 474.116 | 0.00±0.00 |
| Medioresinol | Lignans and Coumarins | C_21_H_24_O_7_ | 388.152 | 0.00±0.00 |
| Secoisolariciresinol | Others | C_20_H_26_O_6_ | 362.172 | 0.00±0.00 |
| L-Ascorbic acid (Vitamin C) | Others | C_6_H_8_O_6_ | 176.032 | 0.37±0.02 |
| Arecatannin A1 | Tannins | C_60_H_50_O_24_ | 1154.269 | 0.05±0.01 |
| Cinnamtannin B2 | Tannins | C_60_H_50_O_24_ | 1154.269 | 2.13±0.00 |
| Acetoxyacetic acid | Organic acids | C_4_H_6_O_4_ | 118.027 | 0.00±0.00 |
| 3-hydroxybenzaldehyde | Organic acids | C_7_H_6_O_2_ | 122.037 | 0.00±0.00 |
| Urocanic acid | Organic acids | C_6_H_6_N_2_O_2_ | 138.043 | 0.01±0.00 |
| Sebacate | Organic acids | C_10_H_18_O_4_ | 202.121 | 0.00±0.00 |
| 4-Oxo-9Z,11Z,13E,15E-Octadecatetraenoic Acid | Lipids | C_18_H_26_O_3_ | 290.188 | 0.01±0.00 |
| Rhododendrol | Others | C_10_H_14_O_2_ | 166.099 | 0.02±0.00 |
| Salidroside | Others | C_14_H_20_O_7_ | 300.121 | 0.00±0.00 |
| Methyl salicylate-2-O-glucoside | Others | C_14_H_18_O_8_ | 314.1 | 0.12±0.00 |
| Koaburaside | Others | C_14_H_20_O_9_ | 332.111 | 0.00±0.00 |
| 4-O-(6'-O-Glucosylcaffeoylglucosylferuloyl)-4-hydroxybenzyl alcohol | Others | C_38_H_42_O_18_ | 786.238 | 0.01±0.00 |
