# Supplementary material for: Analysis of the utilization value of different tissues of Taxus×Media based on metabolomics and antioxidant activity
Source: BMC Plant Biol. 2023 May 29;23:285. doi: 10.1186/s12870-023-04308-6 (PMC10226233; doi:10.1186/s12870-023-04308-6)
Supplement: Supplementary file 4 — Supplementary Material 4 [file 12870_2023_4308_MOESM4_ESM.docx]

**Table S3. The differential metabolites of all samples**

| Compounds | Molecular Weight (Da) | Formula | Relative percentage (%) | | | |
| --- | --- | --- | --- | --- | --- | --- |
|  |  |  | TB | TFL | TS | TA |
| Amino acids and their derivatives | | | | | | |
| L-Norleucine | 131.095 | C_6_H_13_NO_2_ | 0.98±0.00 | 2.69±0.00 | 4.29±0.00 | 4.64±0.00 |
| L-Isoleucine | 131.095 | C_6_H_13_NO_2_ | 0.98±0.00 | 2.72±0.00 | 4.21±0.00 | 4.62±0.00 |
| L-Leucine | 131.095 | C_6_H_13_NO_2_ | 0.96±0.00 | 2.66±0.00 | 4.22±0.00 | 4.61±0.00 |
| L-Tyrosine | 181.074 | C_9_H_11_NO_3_ | 0.12±0.00 | 1.29±0.00 | 1.01±0.00 | 3.32±0.00 |
| N-Acetyl-L-Tryptophan | 246.1 | C_13_H_14_N_2_O_3_ | 0±0.00 | 0.14±0.00 | 1.93±0.00 | 2.37±0.00 |
| L-Valine | 117.079 | C_5_H_11_NO_2_ | 0.25±0.00 | 1.05±0.00 | 1.62±0.00 | 2.04±0.00 |
| L-Cyclopentylglycine | 143.095 | C_7_H_13_NO_2_ | 0.13±0.00 | 0.43±0.00 | 1.03±0.00 | 0.97±0.00 |
| L-Glutamic acid | 147.053 | C_5_H_9_NO_4_ | 2.05±0.00 | 0.37±0.00 | 3.01±0.00 | 0.32±0.00 |
| L-Asparagine | 132.054 | C_4_H_8_N_2_O_3_ | 0.13±0.00 | 0.04±0.00 | 0.7±0.00 | 0.01±0.00 |
| L-Lysine-Butanoic Acid | 234.158 | C_10_H_22_N_2_O_4_ | 4.4±0.00 | 0±0.00 | 0±0.00 | 0±0.00 |
| Phenolic acids | | | | | | |
| 4-Hydroxybenzoic acid | 138.032 | C_7_H_6_O_3_ | 4.37±0.00 | 0.36±0.00 | 0.87±0.00 | 0.24±0.00 |
| 3,4-Dihydroxybenzoic acid | 154.027 | C_7_H_6_O_4_ | 7.57±0.00 | 0.06±0.00 | 0.28±0.00 | 0.09±0.00 |
| 2,5-Dihydroxybenzoic acid | 154.027 | C_7_H_6_O_4_ | 3.63±0.00 | 0.03±0.00 | 0.09±0.00 | 0.04±0.00 |
| Salicylic acid | 138.032 | C_7_H_6_O_3_ | 1.15±0.00 | 0±0.00 | 0.01±0.00 | 0±0.00 |
| Flavonoids and their derivatives | | | | | | |
| Kaempferol-3-O-sambubioside | 580.142 | C_26_H_28_O_15_ | 0.17±0.00 | 1.15±0.00 | 1.84±0.00 | 0.48±0.00 |
| Gallocatechin | 306.074 | C_15_H_14_O_7_ | 0.19±0.00 | 2.05±0.00 | 2.97±0.00 | 0.46±0.00 |
| Epigallocatechin | 306.074 | C_15_H_14_O_7_ | 0.09±0.00 | 1.27±0.00 | 1.5±0.00 | 0.25±0.00 |
| Aromadendrin | 288.063 | C_15_H_12_O_6_ | 0.04±0.00 | 1.95±0.00 | 0.21±0.00 | 0.22±0.00 |
| 5,7,3',4',5'-Pentahydroxydihydroflavone | 304.059 | C_15_H_12_O_7_ | 0±0.00 | 0.4±0.00 | 1.14±0.00 | 0.19±0.00 |
| Luteolin-7-O-rutinoside | 594.158 | C_27_H_30_O_15_ | 0.08±0.00 | 1.37±0.00 | 0.16±0.00 | 0.15±0.00 |
| Naringenin | 272.068 | C_15_H_12_O_5_ | 0.02±0.00 | 1.71±0.00 | 0.49±0.00 | 0.11±0.00 |
| Cyanidin-3-O-(2''-O-glucosyl)glucoside | 611.161 | C_27_H_31_O_16_+ | 0.01±0.00 | 0.08±0.00 | 0.6±0.00 | 0.07±0.00 |
| Kaempferol-3-O-rutinoside | 594.158 | C_27_H_30_O_15_ | 0.04±0.00 | 0.84±0.00 | 0.05±0.00 | 0.03±0.00 |
| Quercetin-3-O-rhamnoside | 448.101 | C_21_H_20_O_11_ | 0.27±0.00 | 1.21±0.00 | 0.11±0.00 | 0.03±0.00 |
| Isosalipurposide | 434.121 | C_21_H_22_O_10_ | 0.01±0.00 | 1.12±0.00 | 0.05±0.00 | 0.03±0.00 |
| Gallocatechin 3-O-gallate | 458.085 | C_22_H_18_O_11_ | 2.37±0.00 | 0.14±0.00 | 0.02±0.00 | 0.02±0.00 |
| Amentoflavone | 538.09 | C_30_H_18_O_10_ | 0.02±0.00 | 1.66±0.00 | 0.12±0.00 | 0.02±0.00 |
| Epigallocatechin-3-gallate | 458.085 | C_22_H_18_O_11_ | 1.91±0.00 | 0.17±0.00 | 0.02±0.00 | 0.02±0.00 |
| Avicularin | 434.085 | C_20_H_18_O_11_ | 0.17±0.00 | 3.64±0.00 | 0.06±0.00 | 0.02±0.00 |
| Morin-3-O-lyxoside | 434.085 | C_20_H_18_O_11_ | 0.1±0.00 | 2.48±0.00 | 0.04±0.00 | 0.01±0.00 |
| Galangin | 270.053 | C_15_H_10_O_5_ | 0±0.00 | 1.22±0.00 | 0.01±0.00 | 0.01±0.00 |
| Apigenin-7-O-glucoside | 432.106 | C_21_H_20_O_10_ | 0.01±0.00 | 0.84±0.00 | 0.01±0.00 | 0±0.00 |
| Kaempferol-4'-O-glucoside | 448.101 | C_21_H_20_O_11_ | 0±0.00 | 2.13±0.00 | 0±0.00 | 0±0.00 |
| Saccharides and their derivatives | | | | | | |
| D-Glucose | 180.063 | C_6_H_12_O_6_ | 0.1±0.00 | 0.43±0.00 | 1.13±0.00 | 2.89±0.00 |
| D-Fructose | 180.063 | C_6_H_12_O_6_ | 0.1±0.00 | 0.38±0.00 | 0.96±0.00 | 2.63±0.00 |
| D-Mannose | 180.063 | C_6_H_12_O_6_ | 0.08±0.00 | 0.37±0.00 | 1.14±0.00 | 2.47±0.00 |
| D-Galactose | 180.063 | C_6_H_12_O_6_ | 0±0.00 | 0.19±0.00 | 0.44±0.00 | 1.75±0.00 |
| D-Saccharic acid | 210.038 | C_6_H_10_O_8_ | 0.01±0.00 | 0.16±0.00 | 0.6±0.00 | 0.63±0.00 |
| D-Galactaric acid | 210.038 | C_6_H_10_O_8_ | 0.01±0.00 | 0.17±0.00 | 0.53±0.00 | 0.57±0.00 |
| Gluconic acid | 196.058 | C_6_H_12_O_7_ | 0.02±0.00 | 0.04±0.00 | 1.88±0.00 | 0.2±0.00 |
| Alkaloids | | | | | | |
| Spicataxine | 669.351 | C_37_H_51_NO_10_ | 0.05±0.00 | 1.26±0.00 | 4.4±0.00 | 0.17±0.00 |
| Spicaledonine | 667.335 | C_37_H_49_NO_10_ | 0.01±0.00 | 0.65±0.00 | 1.32±0.00 | 0.14±0.00 |
| Betaine | 117.079 | C_5_H_11_NO_2_ | 3.86±0.00 | 0.01±0.00 | 0.05±0.00 | 0.05±0.00 |
| Organic acids | | | | | | |
| 3-Dehydro-L-Threonic acid | 134.022 | C_4_H_6_O_5_ | 0.01±0.00 | 0.59±0.00 | 0.53±0.00 | 5.16±0.00 |
| Succinic acid | 118.027 | C_4_H_6_O_4_ | 0.2±0.00 | 0.46±0.00 | 1.32±0.00 | 2.73±0.00 |
| Methylmalonic acid | 118.027 | C_4_H_6_O_4_ | 0.18±0.00 | 0.46±0.00 | 1.23±0.00 | 2.66±0.00 |
| Isocitric acid | 192.027 | C_6_H_8_O_7_ | 0.04±0.00 | 0.59±0.00 | 2.21±0.00 | 0.87±0.00 |
| D-Xylonic acid | 166.048 | C_5_H_10_O_6_ | 0.04±0.00 | 0.33±0.00 | 0.95±0.00 | 0.63±0.00 |
| β-Hydroxyisovaleric acid | 118.063 | C_5_H_10_O_3_ | 0.01±0.00 | 0.3±0.00 | 0.66±0.00 | 0.17±0.00 |
| γ-Aminobutyric acid | 103.063 | C_4_H_9_NO_2_ | 0.02±0.00 | 0.07±0.00 | 0.59±0.00 | 0.16±0.00 |
| 2,3-Dihydroxybenzoic acid | 154.027 | C_7_H_6_O_4_ | 8.16±0.00 | 0.07±0.00 | 0.36±0.00 | 0.1±0.00 |
| Malonic acid | 104.06 | C_3_H_4_O_4_ | 2.06±0.00 | 0.04±0.00 | 0.42±0.00 | 0.05±0.00 |
| Shikimic acid | 174.053 | C_7_H_10_O_5_ | 0±0.00 | 0.71±0.00 | 0.01±0.00 | 0.03±0.00 |
| Lipids | | | | | | |
| LysoPE 16:0 | 453.286 | C_21_H_44_NO_7_P | 0.12±0.00 | 0.3±0.00 | 0.24±0.00 | 3.66±0.00 |
| LysoPC 18:1 | 521.348 | C_26_H_52_NO_7_P | 0.49±0.00 | 0.55±0.00 | 1±0.00 | 2.37±0.00 |
| Myristic Acid | 228.209 | C_14_H_28_O_2_ | 0.53±0.00 | 0.28±0.00 | 2.05±0.00 | 1.14±0.00 |
| Elaidic Acid | 282.256 | C_18_H_34_O_2_ | 1.49±0.00 | 0.52±0.00 | 1.37±0.00 | 1.11±0.00 |
| LysoPE 18:1 | 479.301 | C_23_H_46_NO_7_P | 0.05±0.00 | 0.02±0.00 | 0.3±0.00 | 0.59±0.00 |
| Others | | | | | | |
| Adenine | 135.054 | C_5_H_5_N_5_ | 0.11±0.00 | 0.16±0.00 | 0.36±0.00 | 1.37±0.00 |
| 2'-Deoxyinosine-5'-monophosphate | 332.052 | C_10_H_13_N_4_O_7_P | 0.09±0.00 | 0.02±0.00 | 1.22±0.00 | 1.16±0.00 |
| Inositol | 180.063 | C_6_H_12_O_6_ | 0.08±0.00 | 0.36±0.00 | 1.15±0.00 | 2.39±0.00 |
| 2a-(a-M ethylbutyryD)-oxy-5a,7B,10f-triacetoxy-4(20),1 1l-taxad-iene | 546.319 | C_31_H_46_O_8_ | 0.1±0.00 | 1.05±0.00 | 0±0.00 | 0.02±0.00 |
| Dulcitol | 182.079 | C_6_H_14_O_6_ | 1.61±0.00 | 0±0.00 | 0.05±0.00 | 0.02±0.00 |
| Taxuspine B | 622.277 | C_35_H_42_O_10_ | 0.01±0.00 | 0.03±0.00 | 0.97±0.00 | 0.01±0.00 |
| Secoisolariciresinol | 362.172 | C_20_H_26_O_6_ | 0±0.00 | 1±0.00 | 0±0.00 | 0±0.00 |
| Procyanidin B1 | 578.142 | C_30_H_26_O_12_ | 0.14±0.00 | 0.71±0.00 | 0.04±0.00 | 0.04±0.00 |
| 2,5-Dihydroxybenzaldehyde | 138.032 | C_7_H_6_O_3_ | 3.27±0.00 | 0.26±0.00 | 0.63±0.00 | 0.17±0.00 |
| Protocatechualdehyde | 138.032 | C_7_H_6_O_3_ | 2.51±0.00 | 0.22±0.00 | 0.37±0.00 | 0.13±0.00 |
| Dibutyl phthalate | 278.152 | C_16_H_22_O_4_ | 2.66±0.00 | 0.27±0.00 | 0.62±0.00 | 0.31±0.00 |
| 4-Hydroxybenzaldehyde | 122.037 | C_7_H_6_O_2_ | 0.11±0.00 | 1.19±0.00 | 0.01±0.00 | 0.01±0.00 |
